# Fine-Mapping of the *PLCL2* Gene Identifies Candidate Variants Associated With Ischaemic Stroke Risk in Metabolic Syndrome Patients

**DOI:** 10.3389/fneur.2021.743169

**Published:** 2022-01-20

**Authors:** Xiaoya Huang, Qiang Ye, Yanlei Zhang, Yanyan Chen, Jia Li, Jun Sun, Zusen Ye

**Affiliations:** ^1^Department of Neurology, Wenzhou Central Hospital, Dingli Clinical Institute of Wenzhou Medical University, Wenzhou, China; ^2^Department of Neurology, The First Affiliated Hospital of Wenzhou Medical University, Wenzhou, China; ^3^Department of Neurosurgery, Wenzhou Central Hospital, Dingli Clinical Institute of Wenzhou Medical University, Wenzhou, China; ^4^Wenzhou Key Laboratory of Perioperative of Perioperative Medicine, Wenzhou, China

**Keywords:** ischaemic stroke, *PLCL2* gene, polymorphism, metabolic syndrome, prognosis

## Abstract

A genome-wide association study (GWAS) reported *PLCL2* on chromosome 3p24. 3 (rs4618210:A>G) as a novel susceptibility locus for myocardial infarction in the Japanese population. As the most common pathological process, atherosclerosis leads to metabolic syndrome (MetS)-related ischaemic stroke (IS) and myocardial infarction. Hypothesizing that polymorphisms of the *PLCL2* gene might be associated with the onset and prognosis of IS in MetS patients, we performed the following study in a Chinese Han population. A total of 709 cases (patients with MetS plus IS) and 711 controls (patients with MetS) were enrolled. A fine-mapping strategy was adopted to identify tagged single nucleotide polymorphisms (SNPs) of the *PLCL2* gene, and improved multiplex ligation detection reaction (iMLDR) technology was used to genotype the selected SNPs. Logistic regression was used to analyse the values of the selected SNPs for the risk of IS between the cases and controls, adjusting for sex, age, hypertension, dyslipidaemia, hyperglycaemia, smoking and drinking. To compare the mean age of IS onset among different risk score groups, a genetic risk score was constructed for each case. The cumulative risk of IS events in the case group was presented using a cumulative incidence curve. All cases were followed up for 3 months, and functional outcomes were recorded prospectively. Two SNPs (rs4685423 and rs4618210) were significantly related to the risk of IS in MetS patients. For rs4685423, patients who were AA homozygotes were less likely to suffer from IS than C-allele carriers (OR 0.718; 95% CI 0.567–0.909; multivariate-adjusted, *P* = 0.006). For rs4618210, A-allele carriers were less likely to develop IS than patients who were GG homozygotes (OR 0.679; 95% CI 0.548–0.841; multivariate-adjusted, *P* < 0.001). As the genetic risk score increased, the mean age at IS onset decreased (log-rank *P* = 0.010). There was no statistically significant difference in the distribution of the 90-day modified Rankin Scale (mRS) outcomes across the rs4685423 (*P* = 0.319) or rs4618210 polymorphisms (*P* = 0.148). Our findings suggested that genetic polymorphisms of *PLCL2* might be associated with the onset of MetS-related IS. Further studies are warranted to validate our findings in other ethnic populations.

## Introduction

Metabolic syndrome (MetS) encompasses a group of interrelated metabolic, physiological, biochemical and clinical factors, including hypertension, dyslipidaemia, insulin resistance, glucose intolerance and hyperglycaemia (1). These factors accelerate the onset of stroke, type 2 diabetes mellitus, cardiovascular disease, and certain cancers (2, 3). As a complex condition, the development of MetS depends on genetic and environmental (e.g., lifestyle and sex) factors (4–6).

Stroke is a complex disease, and multiple genes interact with environmental risk factors, increasing the risk (7, 8). As the predominant type of stroke, ischaemic stroke (IS) has a multifactorial pathogenesis, with an estimated heritability of ~40% (8, 9). However, identification of the underlying genes has proven challenging. Genome-wide association studys (GWAS) have been remarkably successful in identifying loci that contribute to such complex diseases. More than 1 million single nucleotide polymorphisms (SNPs) spanning the whole genome can be genotyped with microarrays in a single GWAS (10, 11).

A previous GWAS reported that in the *PLCL2* gene, rs4618210 was associated with myocardial infarction (12). Furthermore, in our earlier case-control study, we discovered a significant relationship between rs4618210 and rs4685423 and susceptibility to large artery atherosclerotic stroke (13). Because atherosclerosis is the most common pathological change in stroke and myocardial infarction, we hypothesized that associations between *PLCL2* gene polymorphisms and IS may exist in MetS patients. Therefore, based on previous GWASs, we used a fine-mapping strategy to determine the relationship between *PLCL2* gene polymorphisms and the onset and short-term functional outcome of IS in MetS patients in a Chinese Han population in this investigation. Considering that age is a major risk factor in IS development, we analyzed the effects of the variants in *PLCL2* on the mean age of IS onset.

## Materials and Methods

### Ethics Statement

The institutional review board of the participating hospitals approved all study documents and procedures. Informed consent was provided by participants or by relatives as legally required, in accordance with the guidelines of the Helsinki Declaration.

### Study Subjects

Cases were identified from the Wenzhou Stroke Registry Programme (WSRP) between April 2014 and December 2018. The details of the WSRP have been described previously (14). The patients were enrolled if they (a) were of Chinese Han nationality; (b) had their first-ever IS with large-artery atherosclerosis or small-artery occlusion according to the TOAST (Trial of Org 10172 in Acute Stroke Treat-ment) criteria (15); (c) were hospitalized within 7 days; (d) were diagnosed with MetS; and (e) were older than 18 years.

To reduce the influence of sex and age, the cases and the controls were matched for age and sex using pair matching. The age of the controls was increased or decreased within a 2-year range compared with the age of the cases. Controls were local residents who underwent physical examinations in hospitals. For the controls, inclusion criteria included (a) Chinese Han nationality; (b) a diagnosis of MetS; (c) no history of stroke or cardiovascular diseases; and (d) age over 18 years.

By asking for medical history or inquiring about the patient's previous written medical record, subjects with autoimmune and inflammatory disorders, severe heart, kidney, or liver dysfunction or malignancies were excluded from this study.

### MetS Definition

MetS was defined according to the revised criteria of the AHA/NHLBI statement (16). The data on waist circumference were not available in the current investigation; thus, BMI ≥ 25 was adopted as a proxy for central obesity (17). When ≥3 of the following criteria were met in a subject, MetS was diagnosed: (a) BMI ≥ 25; (b) blood pressure ≥ 130/85 mmHg or antihypertensive medication use; (c) triglyceride level ≥ 1.70 mmol/L or lipid-lowering medication use; (d) high-density lipoprotein cholesterol level <1.03 mmol/L in men and <1.30 mmol/L in women or medication use; and (e) fasting plasma glucose level ≥ 5.6 mmol/L or antidiabetic medication use. Metabolic syndrome score (MMS) was used to evaluate the severity of the metabolic syndrome (18).

### Isolation of DNA and Genotyping

Genomic DNA was extracted from peripheral blood with a Magen DNA isolation Kit (China). Improved multiplex ligation detection reaction (iMLDR) technology was adopted for genotyping *PLCL2* gene polymorphisms (13). For quality control, ~10% of samples were randomly subjected to repeated genotyping, resulting in 100% concordance. Shanghai Genesky Biotechnology Company (Center for Human Genetics Research) supported the entire experimental course according to standard experimental procedures.

### Fine-Mapping Strategy

A fine-mapping strategy was used to identify the tagging SNPs (tSNPs). Briefly, we used HapMap release 27 of merged phase III (http://www.hapmap.org) to identify a linkage disequilibrium (LD) block of 157.5 kb in the *PLCL2* gene (chr3: 16 949 586..17 107 089) in Han Chinese individuals and then used Haploview software (version 4.2; USA) to analyze the fine-mapping of the tSNPs based on their possibility of tagging the surrounding variants. For the selected SNPs, the inclusion criteria were as follows: (a) a minor allele frequency (MAF) > 0.05, (b) a *P*-value for Hardy-Weinberg equilibrium (HWE) test > 0.05, (c) linkage disequilibrium as assessed by *r*^2^ ≥ 0.8, and (d) overall rate of genotyping ≥ 75%. Based on the previous study, rs4618210 was also included (12). As a result, a total of 8 SNPs were selected for this study (Supplementary Table 1).

### Follow-Up and Outcome

After the index stroke, the cases were prospectively followed up for 3 months via telephone interview or clinical visit. Follow-up was performed by trained physicians who were blinded to the baseline data. The modified Rankin Scale (mRS) was used to assess the functional outcome, and the mRS score at 3 months after stroke onset was tracked.

### Statistical Analysis

Baseline characteristics and clinical features were compared between the cases and controls. The chi-square test was used to analyse the distributions of enumeration data, and the independent samples *t*-test was used to assess measurement data with a normal distribution. HWE was used to evaluate the genotypic distributions of the SNPs in the controls. The values of the selected SNPs for indicating the risk of IS were evaluated using logistic regression between the cases and the controls. The variables adjusted for included sex, age, hypertension, dyslipidaemia, hyperglycaemia, smoking and drinking. In logistic regression analysis, all the selected SNPs were assessed by three genetic models: a co-dominant model, a dominant model and a recessive model. Bonferroni correction was performed to control the false positive error rate in the multiple comparisons, and a *P*-value < 0.05/24 (0.002) was deemed to be statistically significant. Power and Sample Size Program version 3.1.2 was used to estimate the power analyses. Because humans are diploid, the sample size (allele) was doubled in both the case and control groups. A type I error probability of 0.05 was used to estimate the power. One-way ANOVA was conducted to assess the mean age of IS onset for the variants of the selected SNPs. To analyse the mean age of IS onset among different risk score groups, a genetic risk score was constructed for each IS patient. The cumulative risk of IS events according to the variants of the selected SNPs in the cases is presented with a cumulative incidence curve, and comparisons were performed using the log-rank test. Event-free survival time was defined as the time from birth until first-ever IS. All analyses were performed by IBM SPSS Statistics (Version 20.0; IBM Corporation, USA) and two-sided values of *P* < 0.05 were deemed statistically significant.

## Results

### Baseline Characteristics and Clinical Features of the Study Subjects

In the present study, 2 patients refused to provide blood samples. Finally, a total of 709 eligible patients with IS plus MetS and 711 eligible patients with MetS only were recruited in the current investigation. The baseline characteristics and clinical features of the participants are summarized in Table 1. The cases and controls were comparable in both age (*P* = 0.909) and sex (*P* = 0.917). The cases had a higher prevalence of dyslipidaemia (*P* = 0.002) and hypertension (*P* = 0.001) than the controls. Furthermore, the admission systolic blood pressure (*P* = 0.020), diastolic blood pressure (*P* = 0.003) and fasting glucose level (*P* = 0.009) were much higher in the cases than in the controls.

**Table 1:** Baseline characteristics of participants.

**Characteristic**	**IS plus MetS (*n* = 709)**	**MetS alone (*n* = 711)**	**Chi-square or *t*-test**	***P*-value**
Age ≥ 65 years, *n* (%)	403 (56.84)	402 (56.54)	0.013	0.909
Male, *n* (%)	365 (51.48)	368 (51.76)	0.011	0.917
Hypertension, *n* (%)	515 (72.64)	457 (64.28)	11.494	0.001^*^
Hyperglycaemia, *n* (%)	308 (43.44)	288 (40.51)	1.256	0.262
Dyslipidaemia, *n* (%)	295 (41.61)	240 (33.76)	9.323	0.002^*^
Smoking, *n* (%)	242 (34.13)	229 (32.21)	0.593	0.441
Drinking, *n* (%)	229 (32.29)	221 (31.08)	0.242	0.622
BMI, (kg/m^2^)	25.22 ± 2.09	25.29 ± 2.05	0.683	0.495
Fasting glucose, (mmol/L)	7.19 ± 3.27	6.79 ± 2.45	2.603	0.009^*^
SBP, (mm Hg)	142.82 ± 18.64	140.56 ± 17.92	2.325	0.020^*^
DBP, (mm Hg)	81.62 ± 11.76	79.83 ± 10.76	2.998	0.003^*^
Triglyceride, (mmol/L)	1.98 ± 1.04	1.99 ± 0.95	0.246	0.806
HDL-C, (mmol/L)	1.13 ± 0.40	1.11 ± 0.36	1.139	0.255
MSS, *n* (%)			2.489	0.115
3	232 (32.72)	261 (36.71)		
≥4	477 (67.28)	450 (63.29)		

*IS, ischaemic stroke; MetS, metabolic syndrome; BMI, body mass index; SBP, systolic blood pressure; DBP, diastolic blood pressure; HDL-C, high-density lipoprotein cholesterol; MSS, Metabolic syndrome score*.

* *P-value < 0.05 was considered statistically significant*.

### *PLCL2* Genetic Variation and Its Association With IS in MetS Patients

Table 2 shows the effect of *PLCL2* genetic variation on the risk of IS in MetS patients. In the crude model, 2 SNPs (rs4685423 and rs4618210) were found to be significantly related to the risk of IS in MetS patients (*P* < 0.002) after Bonferroni correction. Regarding rs4685423, patients with the AA genotype were less likely to have IS than patients with the CC or CA genotype (recessive model, OR 0.684; 95% CI 0.546–0.856; *P* = 0.001). Regarding rs4618210, patients with the AA or GA genotype were less likely to suffer from IS than patients with the GG genotype (dominant model, OR 0.641; 95% CI 0.519–0.791; *P* < 0.001). In the multivariate model, after adjusting for potential confounders, rs4685423 (recessive model, multivariate-adjusted, *P* = 0.006) and rs4618210 (dominant model, multivariate-adjusted, *P* < 0.001) were still significantly associated with the risk of IS in MetS patients.

**Table 2:** Association between *PLCL2* and risk of IS plus MetS.

**Genetic models**	**SNPs**	**Crude model**			**Multivariate model^a^**		
		**OR**	**95% CI**	***P*-value**	**OR**	**95% CI**	***P*-value**
Co-dominant	rs6769249						
	GG	1.000			1.000		
	GA	1.365	1.019–1.827	0.037	1.441	1.069–1.943	0.017
	AA	1.051	0.211–5.230	0.951	1.010	0.200–5.089	0.991
	rs12233492						
	CC	1.000			1.000		
	CT	1.130	0.905–1.412	0.280	1.073	0.847–1.359	0.562
	TT	1.311	0.930–1.848	0.123	1.224	0.845–1.774	0.285
	rs7616589						
	TT	1.000			1.000		
	TC	1.257	0.901–1.753	0.177	1.309	0.934–1.834	0.118
	CC	0.513	0.094–2.810	0.442	0.481	0.087–2.671	0.403
	rs7612044						
	GG	1.000			1.000		
	GC	0.916	0.657–1.277	0.604	0.952	0.674–1.343	0.778
	CC	0.811	0.579–1.135	0.222	0.890	0.619–1.279	0.528
	rs6789316						
	AA	1.000			1.000		
	AT	1.078	0.860–1.351	0.514	1.061	0.837–1.346	0.622
	TT	0.987	0.561–1.735	0.964	1.098	0.618–1.952	0.750
	rs12630448						
	TT	1.000			1.000		
	TG	0.759	0.595–0.966	0.025	0.727	0.567–0.932	0.012
	GG	1.102	0.623–1.948	0.738	1.107	0.618–1.982	0.733
	rs4685423						
	CC	1.000			1.000		
	CA	0.780	0.582–1.044	0.095	0.794	0.590–1.070	0.130
	AA	0.568	0.415–0.777	<0.001	0.601	0.432–0.836	0.003
	rs4618210						
	GG	1.000			1.000		
	GA	0.650	0.518–0.815	<0.001	0.672	0.534–0.845	0.001
	AA	0.615	0.449–0.844	0.003	0.700	0.507–0.968	0.031
Dominant	rs6769249	1.355	1.015–1.808	0.039	1.426	1.062–1.916	0.018
	rs12233492	1.165	0.943–1.439	0.158	1.097	0.872–1.379	0.430
	rs7616589	1.217	0.878–1.687	0.238	1.262	0.906–1.758	0.169
	rs7612044	0.865	0.631–1.185	0.366	0.928	0.665–1.293	0.658
	rs6789316	1.068	0.859–1.328	0.552	1.066	0.849–1.338	0.585
	rs12630448	0.794	0.631–1.000	0.050	0.765	0.603–0.970	0.027
	rs4685423	0.690	0.523–0.911	0.009	0.724	0.543–0.964	0.027
	rs4618210	0.641	0.519–0.791	<0.001	0.679	0.548–0.841	<0.001
Recessive	rs6769249	1.003	0.202–4.985	0.997	0.961	0.191–4.841	0.961
	rs12233492	1.228	0.889–1.695	0.212	1.171	0.834–1.644	0.362
	rs7616589	0.500	0.091–2.739	0.424	0.466	0.084–2.587	0.383
	rs7612044	0.869	0.703–1.073	0.193	0.927	0.738–1.163	0.511
	rs6789316	0.963	0.551–1.684	0.895	1.080	0.610–1.912	0.793
	rs12630448	1.184	0.672–2.086	0.558	1.213	0.681–2.162	0.513
	rs4685423	0.684	0.546–0.856	0.001	0.718	0.567–0.909	0.006
	rs4618210	0.755	0.561–1.016	0.064	0.847	0.625–1.149	0.287

*IS, ischaemic stroke; MetS, metabolic syndrome; SNP, single nucleotide polymorphism; OR, odds ratio; CI, confidence interval*.

a *Adjusted for age, sex, hypertension, hyperglycaemia, dyslipidaemia, smoking, and drinking*.

In the power analyses, the ORs for rs4685423 and rs4618210 were 0.759 and 0.727, respectively. Based on the sample size, the statistical power of the present study achieved 95.30 and 98.10% for rs4685423 and rs4618210, respectively.

### Effects of the Two Variants in *PLCL2* on the Mean Age of IS Onset

**Figure 1:**
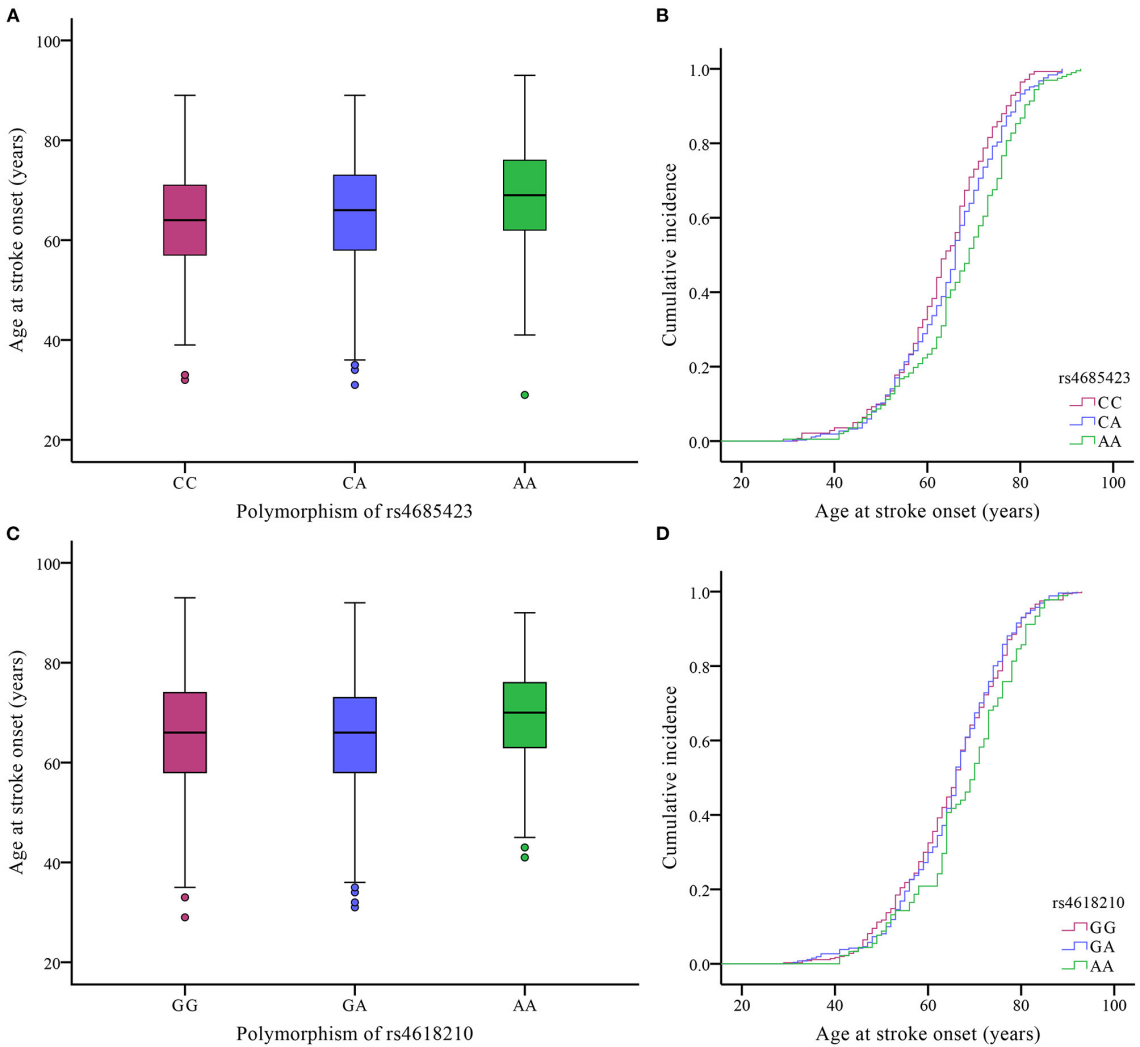
Effects of the polymorphisms of rs4685423 and rs4618210 on the age of ischaemic stroke onset in the cases. **(A)** Box plot of age at onset among ischaemic stroke patients with the CC genotype, CA genotype and AA genotype in rs4685423 (*P* = 0.003); **(B)** Cumulative incidence curve of ischaemic stroke patients with the polymorphisms of rs4685423 (log-rank *P* < 0.001); **(C)** Box plot of age at onset among ischaemic stroke patients with the GG genotype, GA genotype and AA genotype in rs4618210 (*P* = 0.079); **(D)** Cumulative incidence curve of ischaemic stroke patients with the polymorphisms of rs4618210 (log-rank *P* = 0.113).

We evaluated whether the rs4685423 and rs4618210 polymorphisms affected the age of IS onset in the cases. For rs4685423, patients with the AA genotype had a much higher mean age of IS onset than patients with the CC or CA genotype (*P* = 0.003; Figure 1A). The mean ages of onset for the CC genotype, CA genotype and AA genotype were 63.61 ± 10.84, 65.15 ± 11.16 and 67.65 ± 11.82, respectively (*P* = 0.003; Table 3). The rs4685423 polymorphism was significantly related to the mean age of IS onset (log-rank *P* < 0.001; Figure 1B). The mean ages of onset for the GG, GA and AA genotypes in rs4618210 were 65.06 ± 11.50, 65.33 ± 11.13, and 68.02 ± 11.27, respectively (*P* = 0.079; Figure 1C; Table 3). No association was found between the rs4618210 polymorphism and age of stroke onset (log-rank *P* = 0.113; Figure 1D).

**Table 3:** Association between the rs4685423 and rs4618210 polymorphisms and genetic risk score with mean age at ischaemic stroke onset.

**Variants**	**Mean age in different groups (** ***N*** **/mean age)**			**Comparison of mean age in different groups (** ***P*** **-value)**			
rs4685423	CC	CA	AA	Overall	CC vs. CA	CC vs. AA	CA vs. AA
	141/63.61	371/65.15	197/67.65	0.003	0.169	0.001	0.012
rs4618210	GG	GA	AA	Overall	GG vs. GA	GG vs. AA	GA vs. AA
	357/65.06	261/65.33	91/68.02	0.079	0.773	0.026	0.051
Genetic risk score^a^	0	1	2	Overall	0 vs. 1	0 vs. 2	1 vs. 2
	70/69.16	395/65.70	244/64.24	0.006	0.018	0.001	0.114

a *The number represents the numbers of risk genotypes within the combined genotypes; the risk genotypes used for the calculation were rs4685423 CC/CA and rs4618210 GG*.

**Figure 2:**
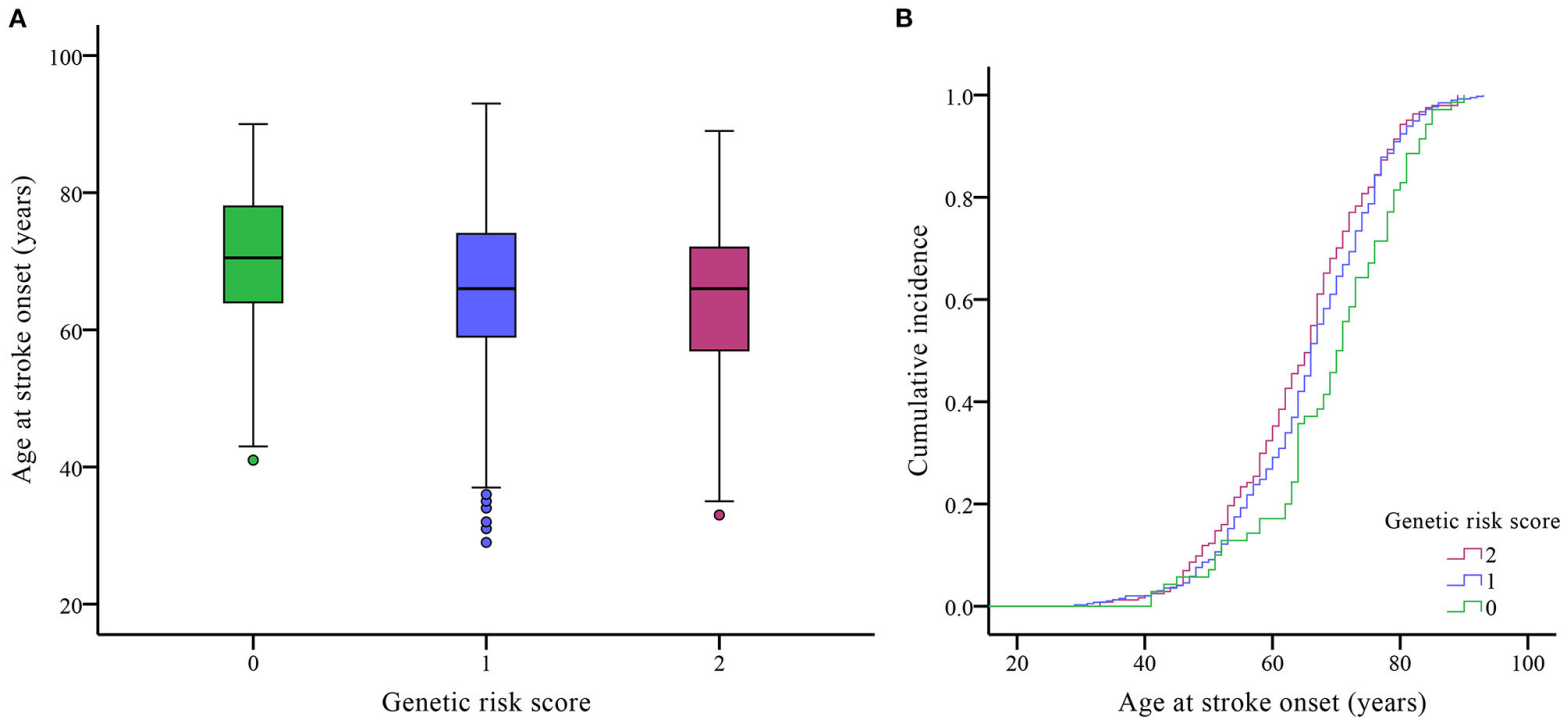
Effects of the genetic risk score on the age of ischaemic stroke onset. **(A)** Box plot of age at onset in three genetic risk score groups (*P* = 0.006). **(B)** Cumulative incidence curve of patients by genetic risk score (log-rank *P* = 0.010).

Because of the potential interactions of the *PLCL2* variants with age at onset, we combined the rs4685423 and rs4618210 polymorphisms according to the number of risk genotypes (i.e., rs4685423 CC/CA and rs4618210 GG). As a result, the mean age was 69.16 ± 11.45 years for those with no risk genotype (rs4685423 AA and rs4618210 GA/AA), 65.70 ± 11.25 years for those with one risk genotype (rs4685423 CC/CA or rs4618210 GG), and 64.24 ± 11.31 years for those with two risk genotypes (rs4685423 CC/CA and rs4618210 GG) (*P* = 0.006; Figure 2A; Table 3). The mean age at IS onset decreased with increasing genetic risk score (log-rank *P* = 0.010; Figure 2B).

### Association Between the Two Variants in *PLCL2* and 3-Month Functional Outcome

After a 3-month follow-up, 9 IS patients with MetS were lost to follow-up. The remaining 700 IS patients with MetS were analyzed for the relationship between the rs4685423 and rs4618210 polymorphisms and 3-month functional outcome.

**Figure 3:**
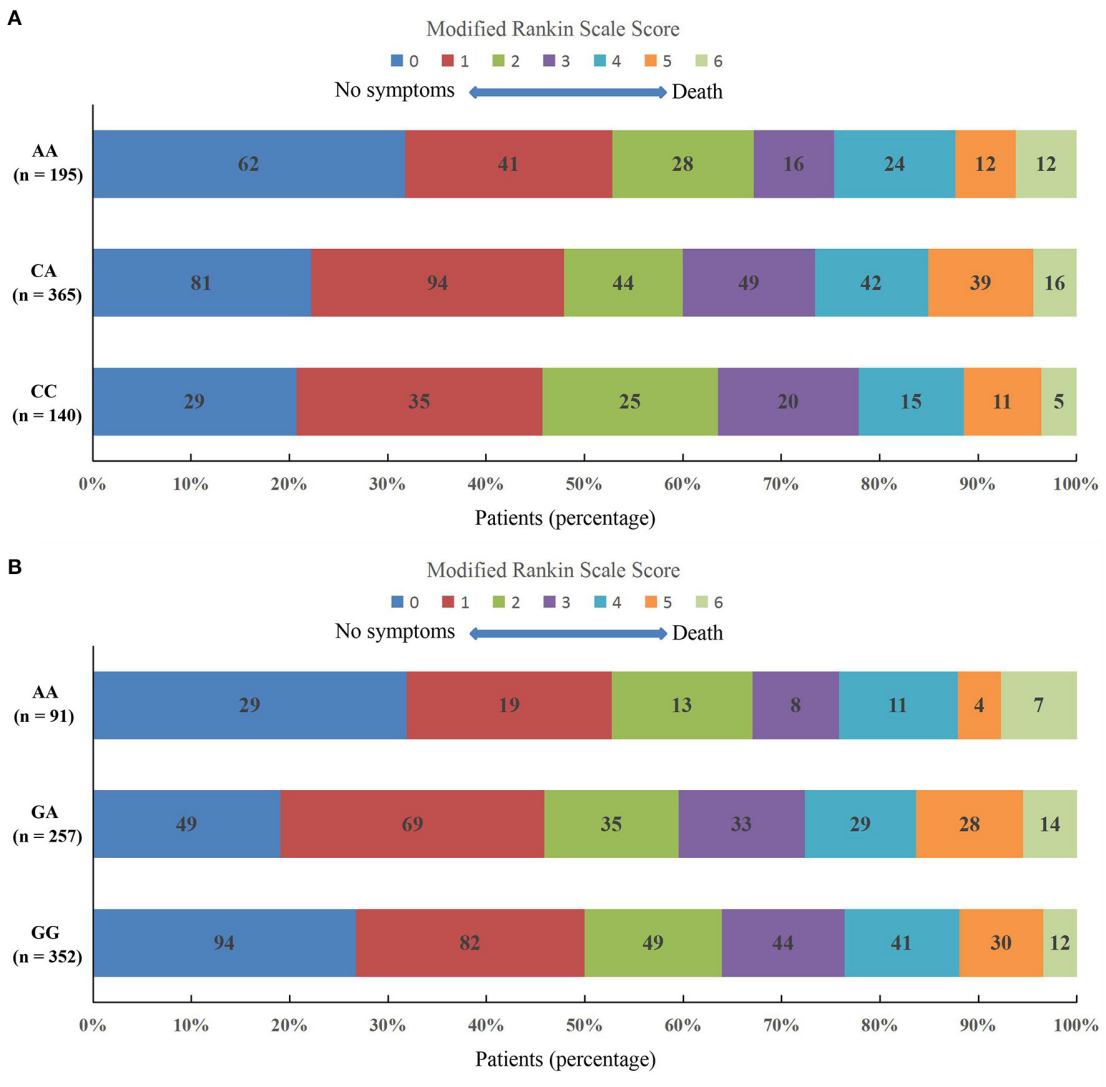
Scores on the modified Rankin Scale at 3 months in ischaemic stroke patients with metabolic syndrome across the polymorphisms of rs4685423 and rs4618210. There was no significantly different shift in the distribution of the mRS scores across the polymorphisms of rs4685423 (**A**, *F* = 1.146, *P* = 0.319) or rs4618210 (**B**, *F* = 1.919, *P* = 0.148).

The mRS scores at 3 months in IS patients with MetS across the rs4685423 and rs4618210 polymorphisms are presented in Figure 3. There was no statistically significant difference in the distribution of the mRS scores across the rs4685423 (Figure 3A; *P* = 0.319) or rs4618210 (Figure 3B; *P* = 0.148) polymorphisms.

## Discussion

In this study, we investigated the relationship of the polymorphisms of the *PLCL2* gene with the onset and short-term prognosis of IS with MetS. The results showed that MetS patients with the rs4685423 AA genotype and the rs4618210 AA/GA genotype had a decreased risk of IS. Notably, the rs4685423 AA genotype was significantly correlated with later age of IS onset. As the genetic risk score increased, the mean age of IS onset decreased. These findings provide insights into the genetic basis of MetS-related IS and suggest a novel therapeutic approach that may be applicable for future stroke prevention.

MetS is a rapidly progressing global socioeconomic problem because of increasing obesity and age (19, 20). It is related to endothelial dysfunction and a pro-inflammatory state that places subjects at a high risk of atherosclerosis. Moreover, data from previous studies showed that MetS elevated the risk of stroke, cardiovascular disease, stroke recurrence, and all-cause mortality (21–23).

As the most common pathological process, atherosclerosis leads to MetS-related IS and cardiovascular diseases. Atherosclerotic plaques are formed of necrotic cores, accumulated modified lipids, calcified regions, leukocytes, inflamed smooth muscle cells, foam and endothelial cells (24). These abovementioned features indicate that the immune system and many components of the vascular and metabolic systems are involved in the atherosclerosis process (25).

Phospholipase C (PLC) proteins are significant intercessors of the calcium-protein kinase C signaling pathway. PLCL2, a novel catalytically inactive PLC-like protein, is expressed in haematopoietic cells. The *PLCL2* gene is located on chromosome 3p24.3 and has been identified as a novel susceptibility site for myocardial infarction in Japanese populations (12). A case-control study in Iran also found rs4618210A>G polymorphism in *PLCL2* gene contribute to MI etiology (26). Experimental data showed (27) that (a) in PLCL2-deficient mice, mature B cells were hyperproliferative in response to B-cell receptor cross-linking and had a more intense T-cell-independent antigen reaction and that (b) in PLCL2-deficient B cells, calcium influx accumulation in nuclei was increased, and extracellular signal-regulated kinase movement was reinforced. All these data indicated that PLCL2 is a passive regulator of immune responses and B-cell receptor signaling. Mature B cells undergo FAS-reconciled apoptosis (28), and *PLCL2* expression is indirectly affected by FAS (29). Thus, we speculate that rs4685423 and rs4618210 may be directly or indirectly associated with the serum level of PLCL2, which is not only related to the management of B-cell maturation, resulting in the formation of atherosclerotic lesions, but also associated with IS in MetS patients. Our speculation is consistent with that of GeneCards (http://www.genecards.org), which supports that *PLCL2* plays a key role in the pathogenesis of atherosclerosis.

In the current study, patients with the rs4685423 AA genotype had a much higher mean age of IS onset than patients with the CC or CA genotype. Furthermore, as the genetic risk score increased, the age of IS onset decreased. The mechanism underlying the associations is unclear, and further investigations are required to determine the effect of this mutation and its connected effects with other mutations on age at IS onset.

The potential limitations of this investigation warrant consideration. First, waist circumference data were not available and we used BMI instead. However, an earlier study reported that both waist circumference and BMI can be used for the prediction of central obesity in Chinese adults (30). Second, all patients in the present study were Han Chinese. As genetic influence is strongly relevant to ethnic background, the results in our study may not compliant with other races and ethnic groups. Third, the serum level of PLCL2 was not tested. Hence, we did not assess the relationship between different genotypes of rs4685423 and rs4618210 and the serum levels of PLCL2, which restricted our analyses. Fourth, the signaling pathway and the function of *PLCL2* in atherosclerosis remain largely unknown. Thus, molecular and cellular experiments should be designed to further elucidate the mechanism involved.

In summary, our findings imply that rs4618210 and rs4685423 of the *PLCL2* gene might contribute to the development of IS in MetS patients. Subsequent investigations are needed to verify whether the *PLCL2* gene can be a target for a novel therapeutic way to prevent stroke.

## Data Availability Statement

The original contributions presented in the study are included in the article/Supplementary Material, further inquiries can be directed to the corresponding authors.

## Ethics Statement

The studies involving human participants were reviewed and approved by Wenzhou Central Hospital, Dingli Clinical Institute of Wenzhou Medical University; The First Affiliated Hospital of Wenzhou Medical University. The patients/participants provided their written informed consent to participate in this study.

## Author Contributions

XH, QY, JS, and ZY designed the study and analyzed the data. XH, QY, YZ, YC, and ZY performed the experiment. XH, YZ, and ZY wrote the manuscript. YC and JL prepared the figure and edited the manuscript. XH, JL, and ZY provided financial support. All authors contributed to the article and approved the submitted version.

## Funding

This study was supported by Zhejiang Provincial Natural Science Foundation – China (LY17H090016 and LQ17H090001), Zhejiang Provincial Medical and Health Science Technology Project – China (2020RC033 and 2018KY122), and Wenzhou Science and Technology Bureau (Y20190131, Y20180135, and Y20170335).

## Conflict of Interest

The authors declare that the research was conducted in the absence of any commercial or financial relationships that could be construed as a potential conflict of interest.

## Publisher's Note

All claims expressed in this article are solely those of the authors and do not necessarily represent those of their affiliated organizations, or those of the publisher, the editors and the reviewers. Any product that may be evaluated in this article, or claim that may be made by its manufacturer, is not guaranteed or endorsed by the publisher.
