# Supplementary material for: Fine-Mapping of the PLCL2 Gene Identifies Candidate Variants Associated With Ischaemic Stroke Risk in Metabolic Syndrome Patients
Source: Front Neurol. 2022 Jan 20;12:743169. doi: 10.3389/fneur.2021.743169 (PMC8810820; doi:10.3389/fneur.2021.743169)
Supplement: Supplementary file 1 [file Table_1.DOC]

**Supplementary table 1** Information of eight selected SNPs of *PLCL2* gene region in a Chinese population.

| SNP | Chromosome | Location on Chromosome | Allele | MAF (CHB)^a^ | *P* value^b^ |
| --- | --- | --- | --- | --- | --- |
| rs6769249 | 3 | 16977623 | G:A | 0.093 | 0.767 |
| rs12233492 | 3 | 17018417 | C:T | 0.465 | 0.400 |
| rs7616589 | 3 | 17023269 | T:C | 0.056 | 0.197 |
| rs7612044 | 3 | 17025997 | G:C | 0.500 | 0.858 |
| rs6789316 | 3 | 17040120 | A:T | 0.237 | 0.966 |
| rs12630448 | 3 | 17061122 | T:G | 0.186 | 0.586 |
| rs4685423 | 3 | 17089924 | C:A | 0.453 | 0.356 |
| rs4618210 | 3 | 17099388 | G:A | 0.419 | 0.087 |

Abbreviations: MAF = minor allele frequency; SNP = single nucleotide polymorphism.

^a^ From both HapMap and dbSNPs databases.

^b^ *P* value for Hardy-Weinberg equilibrium in the controls.
